# Secocrassumol, a *seco*-Cembranoid from the Dongsha Atoll Soft Coral *Lobophytum crassum*

**DOI:** 10.3390/md12126028

**Published:** 2014-12-17

**Authors:** Shi-Yie Cheng, Shang-Kwei Wang, Chang-Yih Duh

**Affiliations:** 1 Department of Life Sciences, National University of Kaohsiung, Kaohsiung 811, Taiwan; E-Mail: shiyie@nuk.edu.tw; 2 Department of Microbiology, Kaohsiung Medical University, Kaohsiung 807, Taiwan; E-Mail: skwang@cc.kmu.edu.tw; 3 Department of Marine Biotechnology and Resources, National Sun Yat-Sen University, Kaohsiung 804, Taiwan; 4 Graduate Institute of Natural Products, Kaohsiung Medical University, Kaohsiung 807, Taiwan

**Keywords:** *Lobophytum crassum*, *seco*-cembranoid, Mosher’s acylation, antiviral activity

## Abstract

Chemical investigations on the Dongsha Atoll soft coral *Lobophytum crassum* led to the purification of a new *seco*-cembranoid, secocrassumol. The structural elucidation was established by extensive NMR, HRESIMS and CD data. The absolute configuration at C-12 was determined as *S* using a modified Mosher’s acylation. Secocrassumol differs from previously known marine *seco*-cembranoid in that it possesses an unprecedented skeleton functionalized at C11-C12 bond cleavage. Secocrassumol showed antiviral activity against human cytomegalovirus (HCMV) with an IC_50_ value of 5.0 μg/mL.

## 1. Introduction

**Figure 1:**
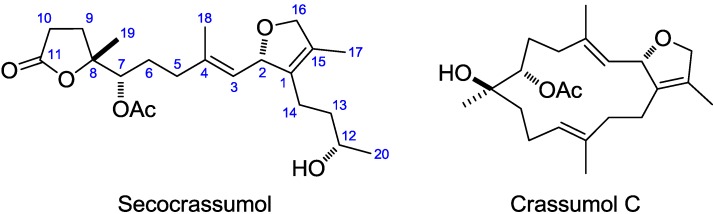
The structure of secocrassumol and crassumol C.

**Scheme 1:**
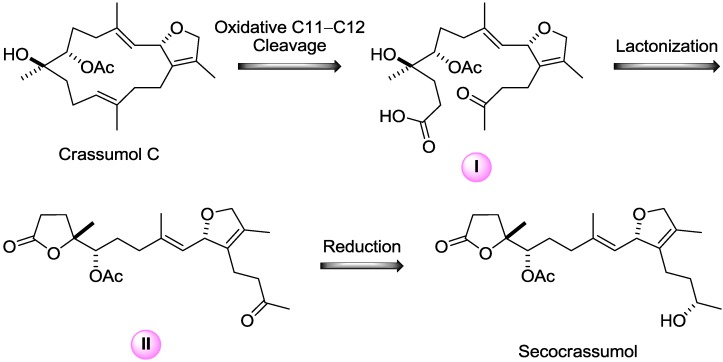
The plausible biosynthetic pathway for formation of secocrassumol.

Marine soft corals have evolved characteristic metabolic and physiological capabilities to produce secondary metabolites that may function in defense, food capture, interference competition, and even possibly the acquisition and selection of symbiotic zooxanthellae [1]. The first cembrane-type diterpenoid was obtained in 1951 from the oleoresin of *Pinus albicaulis* [2]. Marine cembranoids are the representative compounds from soft corals, having been first discovered from gorgonians by the Ciereszko lab in 1960 [3]. For more than 60 years, hundreds of cembranoids possessing almost every structural modification have been reported from virtually all alcyonarians and gorgonians [4]. Prior studies have shown that members of the genus *Lobophytum* produce a rich harvest of cembranoids endowed with diversified macrocyclic skeletons [4,5,6,7,8,9,10,11,12,13,14,15,16,17,18,19,20,21,22,23,24,25]. Previous bioassay results of these metabolites have been shown to exhibit diverse biological activities such as cytotoxicity [11,13,15,16,17,18,19,23], anti-inflammatory properties [20,21,22], antimicrobial activities [20], and HIV-inhibitory activities [17]. During the course of our initial investigation of bioactive metabolites from the soft coral *L. crassum* (von Marenzeller, 1886), six cembranoids (lobocrassolide, lobocrasol, crassumols A–C and 13-acetoxysarcophytoxide) and two á-tocopherols (crassumtocopherols A and B) were discovered and some of these have been shown to possess cytotoxic properties [18,23,24,25]. Our continuing chemical investigations of this organism led to the isolation of secocrassumol (Figure 1). Secocrassumol apparently derives from a cembranoid precursor through cleavage of the C11-C12 bond. The plausible biosynthetic pathway for formation of secocrassumol is postulated in Scheme 1. Furthermore, it was evaluated *in vitro* for cytotoxicity against A-459 (human lung carcinoma), P-388 (mouse lymphocytic leukemia), and HT-29 (human colon adenocarcinoma) cancer cell lines as well as antiviral activity against HCMV (human cytomegalovirus) cells.

## 2. Results and Discussion

**Figure 2:**
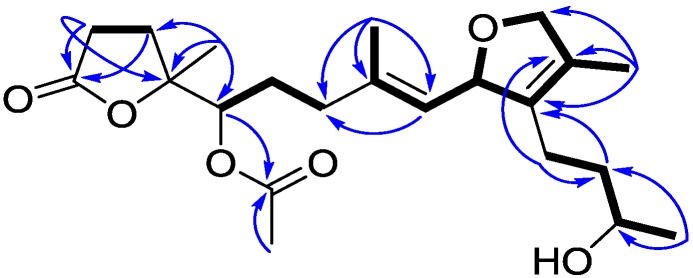
Selected ^1^H–^1^H COSY (▬) and HMBC (**→**) correlations of secocrassumol.

The chromatographic separation of the EtOAc extract (20 g) of the soft coral *L*. *crassum* using Si-60 and ODS gel columns in combination with semi-preparative reversed-phase C_18_ HPLC resulted in the purification of secocrassumol (see Experimental Section), which was obtained as a colorless oil. The HRESIMS exhibited a pseudo molecular ion peak at *m*/*z* 417.2256 [M + Na]^+^ (calcd. for C_22_H_34_O_6_Na, 417.2253), consistent with the molecular formula of C_22_H_34_O_6_, requiring six degrees of unsaturation. The IR spectrum demonstrated a broad absorption band at 3434 cm^−1^ (OH stretching) diagnostic of a secondary hydroxy group, which was associated to C-12 on the basis of the HMBC correlations from Me-20 to C-12 and C-13 (Figure 2). The IR spectrum revealed the presence of an ester (1742 cm^−1^) moiety, which was further identified by the ^1^H·NMR signals at δ_H_ 2.09 (3H, s) and ^13^C NMR signals at δ_C_ 170.4 (qC) and 20.9 (CH_3_) (Table 1). The existence of two quaternary carbons [δ_C_ 133.0 (qC, C-1) and 128.4 (qC, C-15)], an oxygenated methine [δ_H_ 5.09 (dd, 1H, *J* = 9.2, 0.8 Hz) and δ_C_ 84.4 (C-2)] and an oxygenated methylene [δ_H_ 4.54 (dd, 1H, *J* = 11.6, 5.6 Hz) and 4.45 (br d, 1H, *J* = 11.6 Hz); δ_C_ 78.1 CH_2_], as well as the long-range COSY correlations between H-2 and H_2_-16 exhibited the presence of a 2,5-dihydrofuran ring (Figure 2). In addition, the ^13^C NMR signals at δ_C_ 126.0 (CH, C-3) and 138.2 (qC, C-4) assigned a trisubstituted double bond. Although there were no direct HMBC correlations available, the remaining one unsaturation indicated that an oxygen bridge is probably present between a lactone carbonyl carbon [δ_C_ 176.2 (qC, C-11)] and an oxygenated quaternary carbon [δ_C_ 82.6 (qC, C-8)]. This assumption was further confirmed by a strong IR absorption at 1771 cm^−1^ [26]. Comparison to the NMR data reported for crassumol C [24] permitted us to propose the 11,12-secocembranoid structure for secocrassumol.

**Figure 3:**
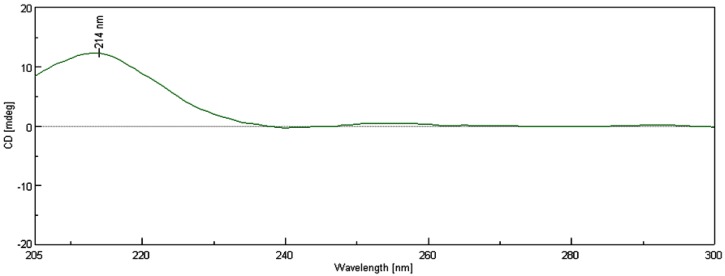
The CD spectrum of secocrassumol.

**Figure 4:**
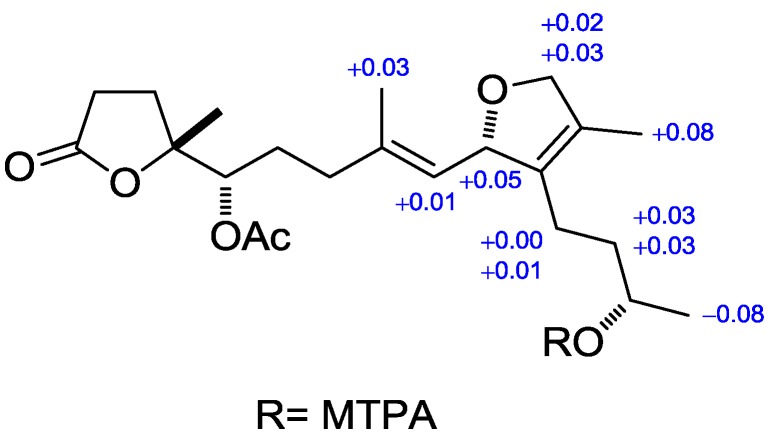
Selected ^1^H·NMR Δδ*_S_*_−*R*_ values in ppm for the *S*- and *R*-MTPA esters of secocrassumol in CDCl_3_.

The geometry of the trisubstituted olefin was assigned as *E* based on the γ-effect of the olefinic methyl signals for C-18 (16.4 ppm) [27] and the NOESY correlations between H-3 and H_2_-5, and H-2 and H_3_-18. The relative configurations of C2, C7 and C8 could not be determined due to the absence of decided NOESY correlations available. Additionally, the failure to crystallize, the limitation of material, and scarcity of sample source make secocrassumol inaccessible to X-ray crystallographic analysis for confirmation of the relative configurations of the aforementioned carbons. Based on biogenic considerations, a 2*S*, 7*S* configuration of secocrassumol was assumed to be identical with that of crassumol C [24]. The circular dichroism (CD) spectrum exhibited a positive Cotton effect around λ_max_ 214 nm due to the γ-lactone (Figure 3). The absolute configuration at C-8 was deduced to be *R* based on its CD data comparable to that of some C11-C12 secocembranoids from the leaves of air-cured Burley tobaccos [26]. The appropriate stereochemistry of secocrassumol was identified by Mosher’s esterification for absolute configuration determination of chiral alcohols [28]. Analysis of the Δδ*_S_*_−*R*_ values according to the Mosher model pointed to an *S* configuration for C-12, because H_2_-13 and H_2_-14 of the (*S*)-MTPA ester were less shielded by the phenyl ring of MTPA products (Figure 4). Accordingly, the structure of secocrassumol was elucidated unambiguously.

**Table 1:** NMR spectroscopic data of secocrassumol *^a^*.

#	^13^C	^1^H	^1^H–^1^H COSY	HMBC (H→C)
1	133.0 (qC) *^b^*			
2	84.4 (CH)	5.42 m	H-3, H-16, H-17	
3	126.0 (CH)	5.09 br·d (9.2, 0.8) *^c^*	H-2, H-18	C-5, C-18
4	138.2 (qC)			
5	35.8 (CH_2_)	2.04 m	H-6	
6	27.6 (CH_2_)	a: 1.94 m	H-5, H-7	
		b: 1.80 m		
7	75.9 (CH)	5.01 dd (10.4, 2.4)	H-6	C-8, C-9, 7-OAc
8	82.6 (qC)			
9	29.9 (CH_2_)	a: 2.22 m	H-10	C-10, C-11, C-19
		b: 1.91 m		
10	28.7 (CH_2_)	2.61 m	H-9	C-8, C-9, C-11
11	176.2 (qC)			
12	68.0 (CH)	3.76 m	H-13, H-20	
13	37.5 (CH_2_)	1.49 m	H-12, H-14	C-1, C-14, C-20
14	21.1 (CH_2_)	a: 2.24 m	H-13	C-1, C-2, C-13
		b: 1.89 m		
15	128.4 (qC)			
16	78.1 (CH_2_)	a: 4.54 dd (11.6, 5.6)	H-2, H-17	C-1
		b: 4.45 br·d (11.6)		
17	10.0 (CH_3_)	1.66 s	H-2, H-16	C-1, C-15, C-16
18	16.4 (CH_3_)	1.76 s	H-3	C-3, C-4, C-5
19	22.5 (CH_3_)	1.40 s		C-7, C-8, C-9
20	23.3 (CH_3_)	1.19 d (6.0)	H-12	C-12, C-13
7-OAc	20.9 (CH_3_)	2.09 s		7-OAc
	170.4 (qC)			

*^a^* Spectra were measured in CDCl_3_ (^1^H, 400 MHz and ^13^C, 100 MHz). *^b^* Multiplicities are deduced by HSQC and DEPT experiments. *^c^ J* values (in Hz) are in parentheses.

Apparently, the co-occurrence of crassumol C within the same organism raises the probability that secocrassumol results from crassumol C. The biosynthetic precursor is transformed into intermediate **I** by oxidative C11-C12 cleavage. The intermediate **I** would undergo an enzymatic lactonization to yield intermediate **II**, which was converted into secocrassumol by reduction (Scheme 1). Purification of secocembranoids is intriguing, especially in light of the previous isolation and characterization of the related metabolites [29,30,31,32,33], suggesting that marine soft corals possess a biodegradable capacity to modify parent cembranoids through simple ring cleavage. The few examples of secocembranoids from soft corals include two C12-C13 analogues from *Sinularia mayi* [29], a C1-C14 secocembranoid from *Eunicea succinea* [30], a C8-C9 secocembranoid (*seco*-sethukarailin) from *Sinularia dissecta* [31], two C2-C3 secocembranoids (caucanolides E and F) from *Pseudopterogorgia bipinnata* [32], two C9-C10 secocembranoids from *Nephthea* sp. [33], and a C11-C12 secocembranoid from *Sinularia flexibilis* [34]. Among these marine secocembranoids already reported in the literature, secocrassumol also represents a secocembrane skeleton functionalized at the C11-C12 bond cleavage.

Preliminary cytotoxic screening revealed that secocrassumol exhibited no discernible cytotoxicity against mouse lymphocytic leukemia (P-388), human lung carcinoma (A-459) as well as human colon adenocarcinoma (HT-29) (ED_50_ > 50 μM). Similarly, the biosynthetic precursor crassumol C was not cytotoxic to P-388, A-549, and HT-29 cells [24]. The anticancer agent mithramycin was used as the positive control and exhibited EC_50_ values of 0.05, 0.06 and 0.07 μM against P-388, A-549 and HT-29 cells, respectively. It was noteworthy to mention that crassumol C did not show anti-HCMV activity (IC_50_ > 50 μg/mL), but secocrassumol exhibited moderate antiviral activity against HCMV cells with an IC_50_ value of 5.0 μg/mL.

## 3. Experimental Section

### 3.1. General Experimental Procedures

Optical rotations were recorded on a JASCO P1020 polarimeter (Tokyo, Japan). CD analysis was performed on a JASCO J-815 spectropolarimeter (Tokyo, Japan). IR and UV spectra were measured on JASCO FT/IR-4100 (Tokyo, Japan) and JASCO V-650 spectrophotometers (Tokyo, Japan), respectively. The NMR spectra were recorded on a Varian 400 MR NMR spectrometer (Santa Clara, CA, USA) at 400 MHz for ^1^H and 100 MHz for ^13^C, respectively. Chemical shifts are expressed in δ (ppm) referring to the solvent peaks δ_H_ 7.27 and δ_C_ 77.0 for CDCl_3_, respectively, and coupling constants are expressed in Hz. ESIMS spectra were recorded by ESI FT-MS on a Bruker APEX II mass spectrometer (Bruker, Bremen, Germany). Silica gel 60 (Merck, Darmstadt, Germany, 230–400 mesh), LiChroprep RP-18 (Merck, Darmstadt, Germany, 40–63 μm) and Sephadex LH-20 (Amersham Pharmacia Biotech., Piscataway, NJ, USA) were used for column chromatography. Precoated silica gel plates (Merck, Darmstadt, Germany, Kieselgel 60 F_254_, 0.25 mm) and precoated RP-18 F_254s_ plates (Merck, Darmstadt, Germany) were used for analytical thin-layer chromatography (TLC) analyses. High-performance liquid chromatography (HPLC) was performed on a Hitachi L-7100 pump (Tokyo, Japan) equipped with a Hitachi L-7400 UV detector (Tokyo, Japan) at 220 nm and an ODS column (Merck, Darmstadt, Germany, Hibar Purospher RP-18e, 5 μm, 250 × 10 mm). *S*-(+)- and *R*-(–)-α-methoxy-α-trifluoromethylphenylacetyl chloride were obtained from ACROS Organics (Geel, Belgium).

### 3.2. Animal Material

Specimens of *L. crassum*, identified by Professor Chang-Feng Dai of the Institute of Oceanography, National Taiwan University (Taipei, Taiwan), were collected from coral reefs offshore from the Dongsha Atoll off Taiwan in April 2007, at a depth of 8 m, and were immediately frozen at −20 °C until further processed for extraction in the laboratory. A voucher specimen (TS-11) has been deposited at the Department of Marine Biotechnology and Resources, National Sun Yat-sen University (Kaohsiung, Taiwan).

### 3.3. Extraction and Isolation

The sliced bodies of *L. crassum* were exhaustively extracted with acetone. The combined extracts were concentrated *in vacuo* (under 35 °C) to obtain a dry crude extract (25 g), which was suspended in water and extracted with EtOAc. The EtOAc-soluble portion was evaporated to dryness *in vacuo* to give a dark brown residue (20 g). The resulting EtOAc residue was subjected to a silica gel chromatography using a stepwise gradient mixture of *n*-hexane–EtOAc–MeOH as elution and separated into 40 fractions. Fraction 20 (223 mg) eluted with *n*-hexane/EtOAc (1:10) was submitted to repeated chromatography over Si-60 gel column using *n*-hexane–EtOAc mixtures of increasing polarity as eluent. Altogether, three subfractions were obtained, of which subfraction 20-3 (142 mg) was followed by column chromatography on ODS column using 53% MeOH in H_2_O to yield a mixture (25 mg). In turn, the mixture was further purified by RP-18 HPLC using an isocratic solvent system of 65% MeOH in H_2_O to give secocrassumol (2 mg).

Secocrassumol: Colorless oil; ${\left[a\right]}_{D}^{25}$ −192 (*c* 0.1, CHCl_3_); IR (KBr) ν_max_ 3434, 2965, 2924, 2857, 1771, 1742, 1649, 1557, 1375, 1232, 1036 cm^−1^; CD (4.80 × 10^−^^4^ M, MeOH) λ_max_ (Δε) 214 (+12.24) nm; ^1^H·NMR (CDCl_3_, 400 MHz) and ^13^C·NMR (CDCl_3_, 100 MHz) data, see Table 1; ESIMS *m*/*z* 417 [M + Na]^+^; HRESIMS *m*/*z* 417.2256 [M + Na]^+^ (calcd. for C_22_H_34_O_6_Na, 417.2253) (Supplementary Figures S1–S7).

### 3.4. Preparation of (R)- and (S)-MTPA Esters of Secocrassumol

Two secocrassumol samples (0.5 mg) were dissolved in pyridine-*d*_5_ (0.6 mL) and allowed to react overnight at room temperature with (*R*)- and (*S*)-MTPA chloride (one drop), affording the (*S*)-MTPA ester (**S**) and (*R*)-MTPA ester (**R**), respectively.

Selected ^1^H-NMR (pyridine-*d*_5_, 400 MHz) of **S**: δ_H_ 7.88–7.61 (5H, m, Ph), 5.70 (1H, m, H-2), 5.35 (1H, d, *J* = 9.2 Hz, H-3), 5.29 (1H, m, H-12), 4.65 (1H, dd, *J* = 11.6, 4.8 Hz, 16a), 4.56 (1H, d, *J* = 11.6 Hz, 16b), 2.70 (1H, t, *J* = 8.8 Hz, H-14a), 2.30 (1H, m, H-14b), 1.87 (1H, m, H-13a), 1.76 (1H, m, H-13b), 2.10 (3H, s, 7-OAc), 1.76 (3H, s, H-18), 1.59 (3H, s, H-17), 1.36 (3H, s, H-19), 1.29 (3H, d, *J* = 6.4 Hz, H-20).

Selected ^1^H-NMR (pyridine-*d*_5_, 400 MHz) of **R**: δ_H_ 7.88–7.61 (5H, m, ph), 5.65 (1H, m, H-2), 5.34 (1H, d, *J* = 9.2 Hz, H-3), 5.30 (1H, m, H-12), 4.63 (1H, dd, *J* = 11.6, 4.8 Hz, 16a), 4.53 (1H, d, *J* = 11.6 Hz, 16b), 2.70 (1H, t, *J* = 8.8 Hz, H-14a), 2.29 (1H, m, H-14b), 1.84 (1H, m, H-13a), 1.73 (1H, m, H-13b), 2.10 (3H, s, 7-OAc), 1.73 (3H, s, H-18), 1.51 (3H, s, H-17), 1.36 (3H, s, H-19), 1.37 (3H, d, *J* = 6.4 Hz, H-20).

### 3.5. Cytotoxicity Assay

Cytotoxicity was determined against P-388 (mouse lymphocytic leukemia), HT-29 (human colon adenocarcinoma), as well as A-549 (human lung epithelial carcinoma) tumor cells using a modification of the MTT colorimetric method according to a previously described procedure [35,36]. The P-388 cell line was kindly provided by John M. Pezzuto, formerly of the Department of Medicinal Chemistry and Pharmacognosy, University of Illinois at Chicago. Additionally, HT-29 and A-549 cell lines were purchased from the American Type Culture Collection (Manassas, VA, USA).

### 3.6. Anti-HCMV Assay

To determine the effects of natural products upon HCMV cytopathic effect (CPE), confluent human embryonic lung (HEL) cells grown in 24-well plates were incubated for 1 h in the presence or absence of various concentrations of tested natural products. Then, cells were infected with HCMV at an input of 1000 pfu (plaque forming units) per well of 24-well dish. Antiviral activity was expressed as IC_50_ (50% inhibitory concentration), or compound concentration required to reduce virus-induced CPE by 50% after seven days, as compared with the untreated control. To monitor the cell growth upon treatment with natural products, an MTT-colorimetric assay was employed [37].

## 4. Conclusions

A new *seco*-cembranoid, designated as secocrassumol, was isolated from the Dongsha Atoll soft coral *Lobophytum crassum*. Secocrassumol differs from the previously known marine *seco*-cembranoid in that it possesses an unprecedented skeleton functionalized at the C11-C12 bond cleavage. Preliminary cytotoxic screening revealed that secocrassumol are not cytotoxic to P-388, A-549, and HT-29 cells. However, secocrassumol showed antiviral activity against HCMV with an IC_50_ value of 5.0 μg/mL.

## Supplementary Files

Supplementary File 1
