# Supplementary material for: Secocrassumol, a seco-Cembranoid from the Dongsha Atoll Soft Coral Lobophytum crassum
Source: Mar Drugs. 2014 Dec 17;12(12):6028–37. doi: 10.3390/md12126028 (PMC4278217; doi:10.3390/md12126028)
Supplement: Supplementary File 1 [file marinedrugs-12-06028-s001.pdf]

Supplementary Information

Figure S1. ^{1}H NMR spectrum (400 MHz) of secocrassumol in $CDCl_3$.

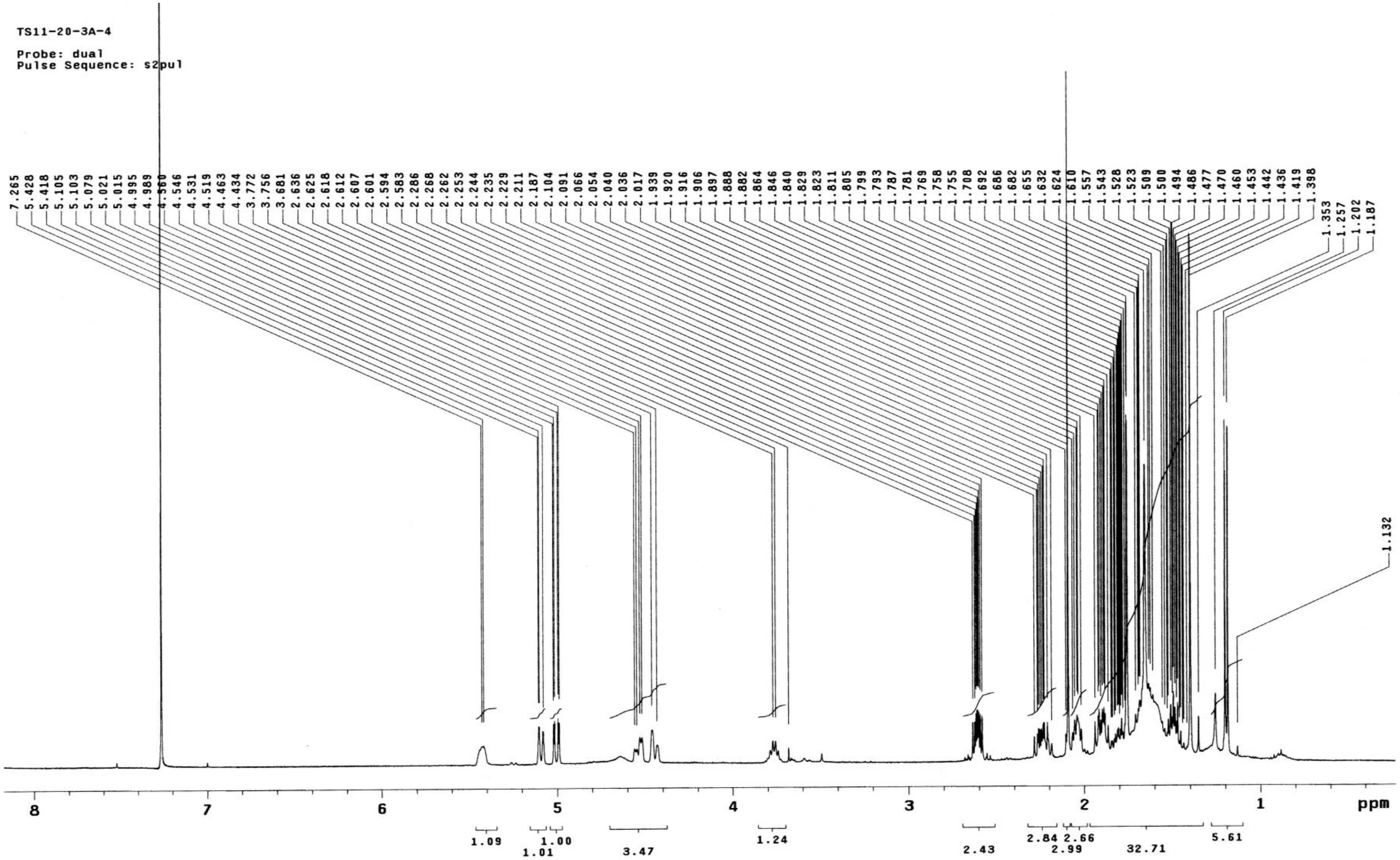

Figure S2. DEPT and ^{13}C NMR spectra (100 MHz) of secocrassumol in $CDCl_3$.

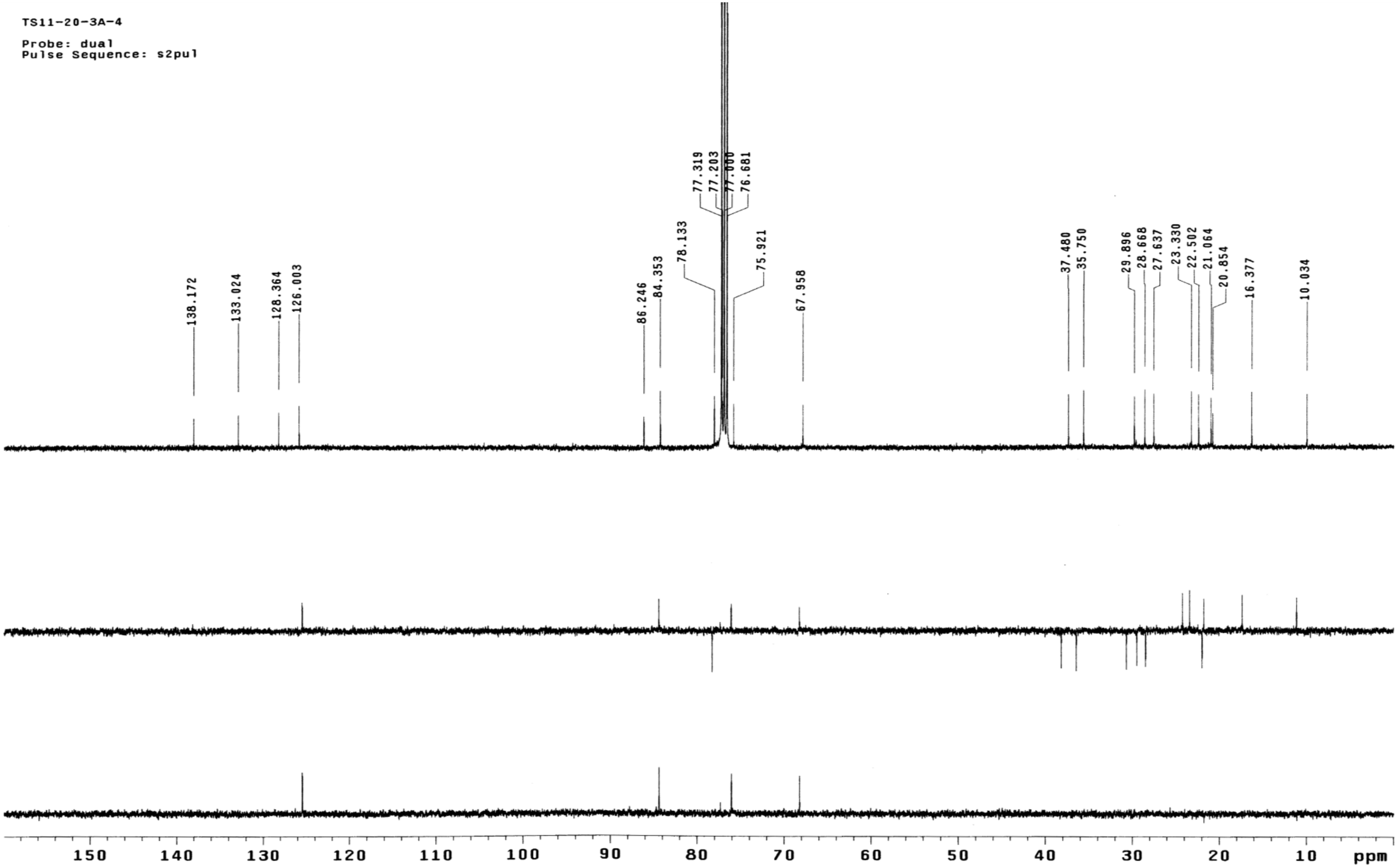

Figure S3. HSQC spectrum (400 MHz) of secocrassumol in $CDCl_3$.

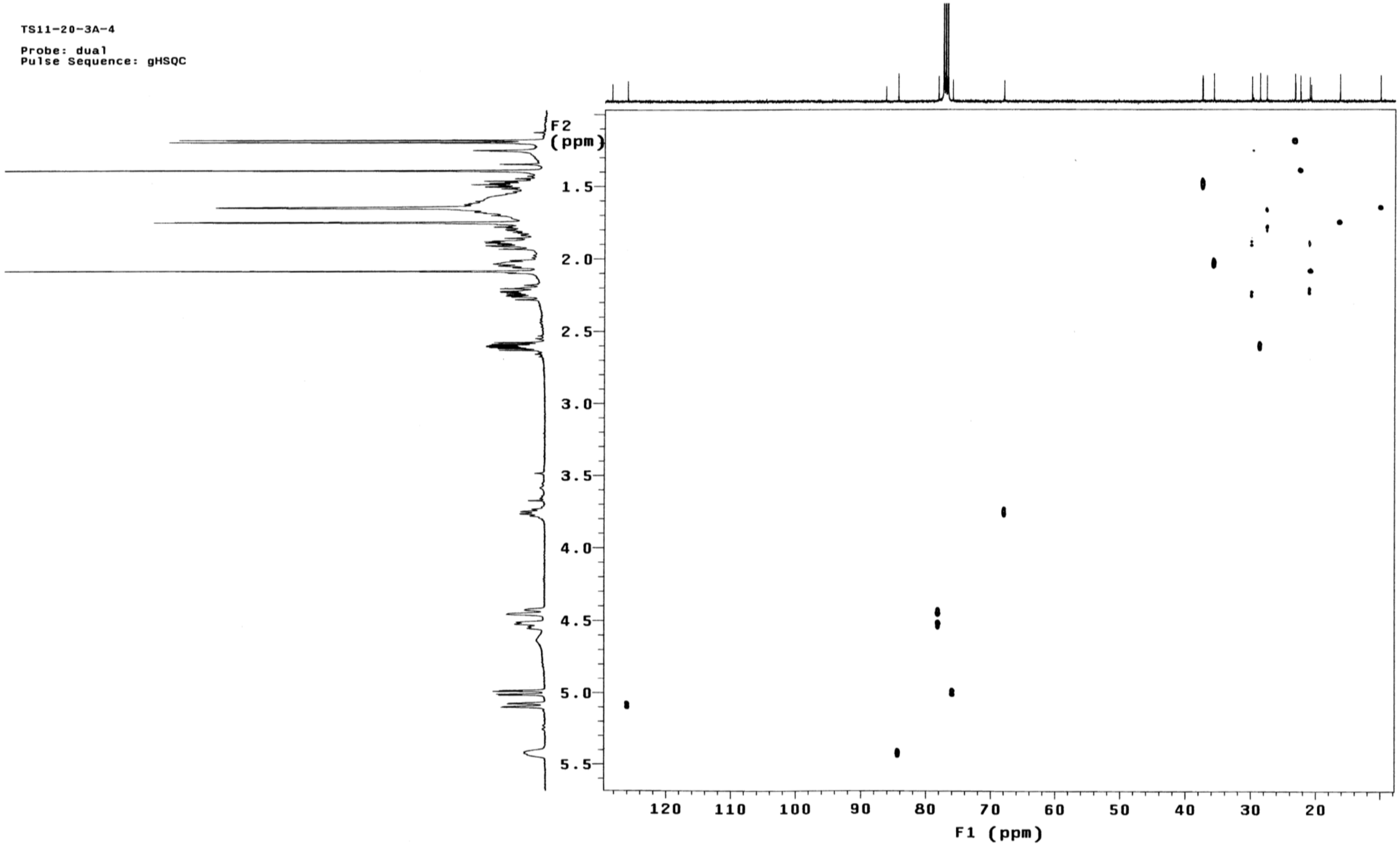

Figure S4. HMBC spectrum (400 MHz) of secocrassumol in $CDCl_3$.

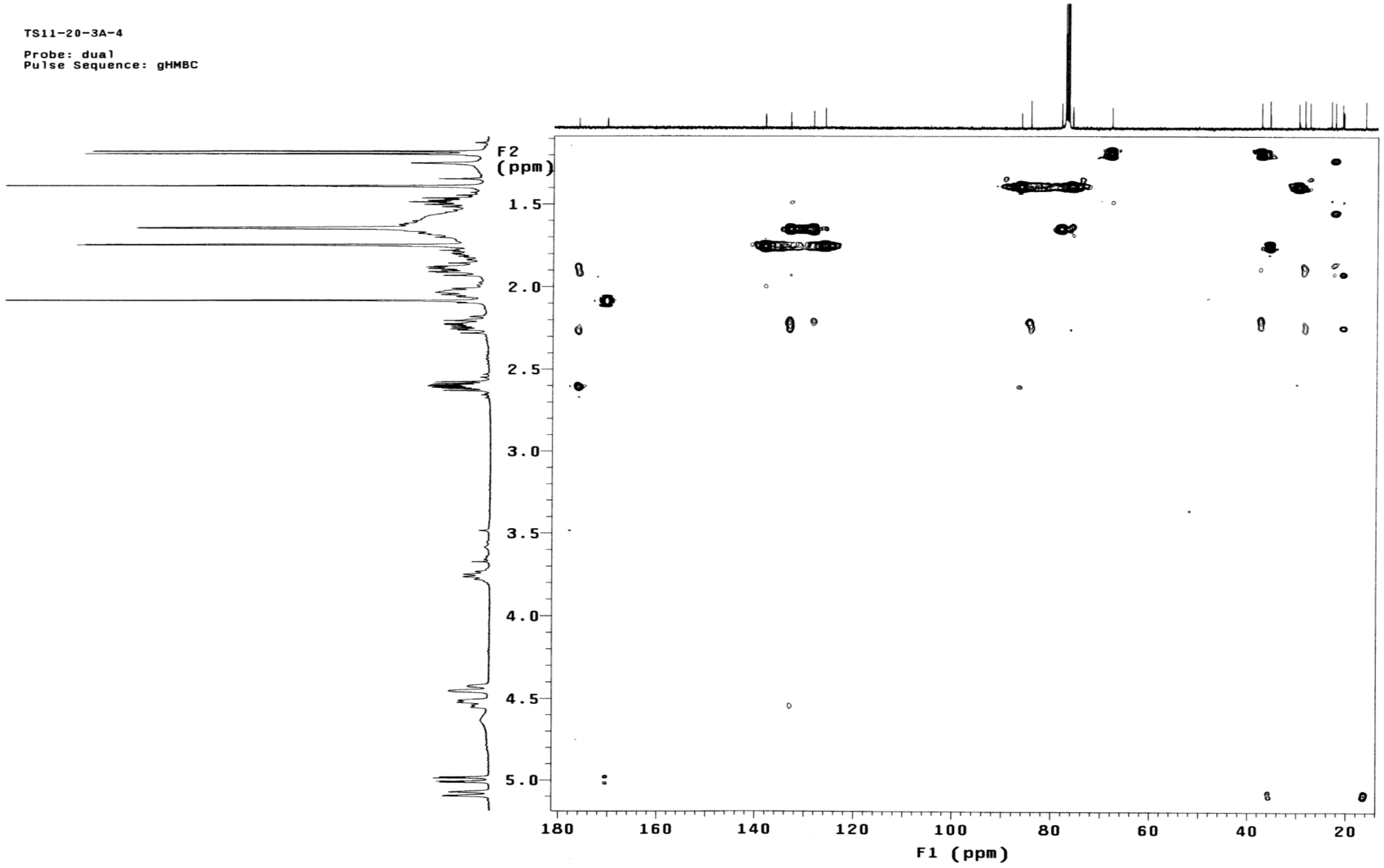

Figure S5. ^1H–^1H COSY spectrum (400 MHz) of secocrassumol in $CDCl_3$.

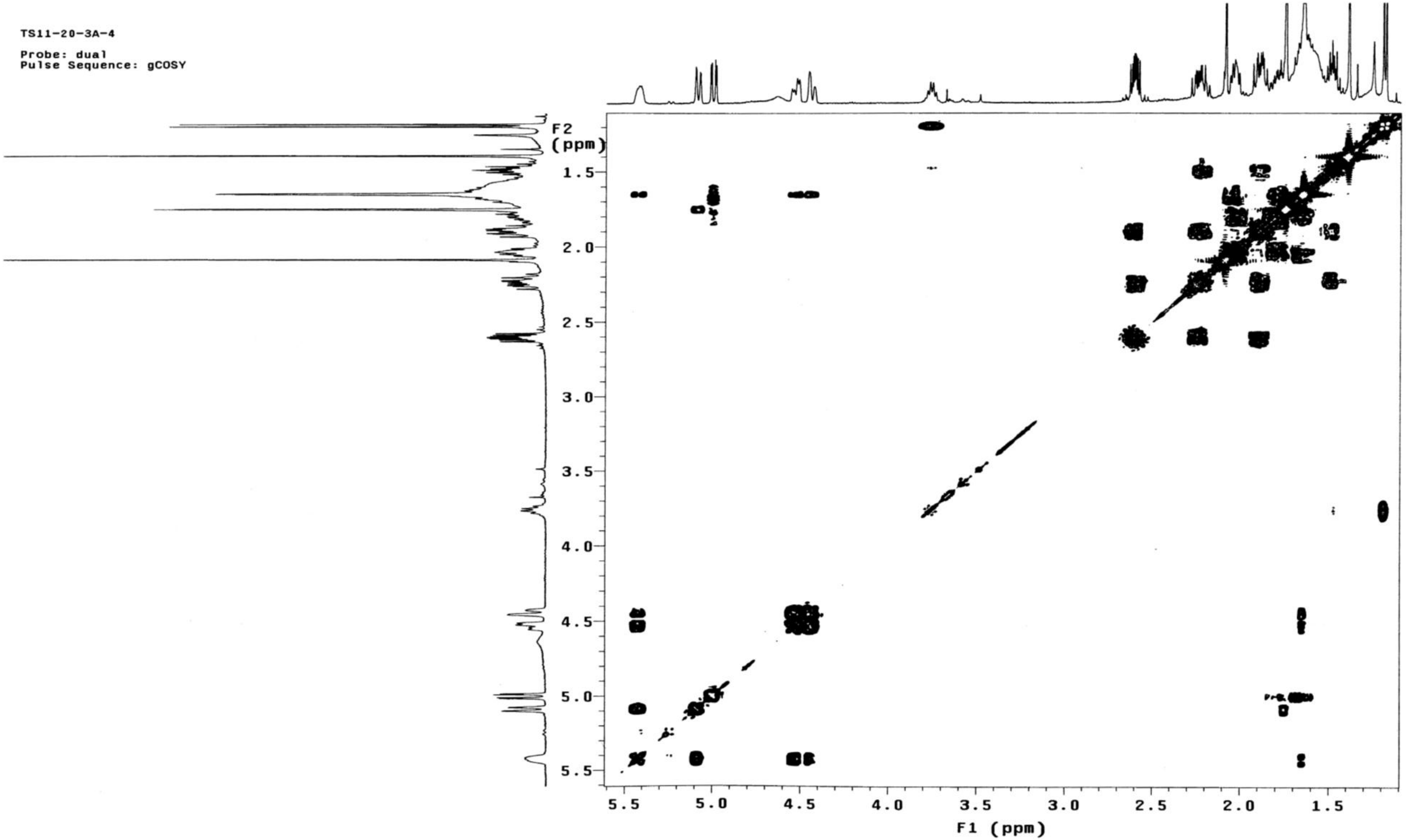

Figure S6. NOESY spectrum (400 MHz) of secocrassumol in $CDCl_3$.

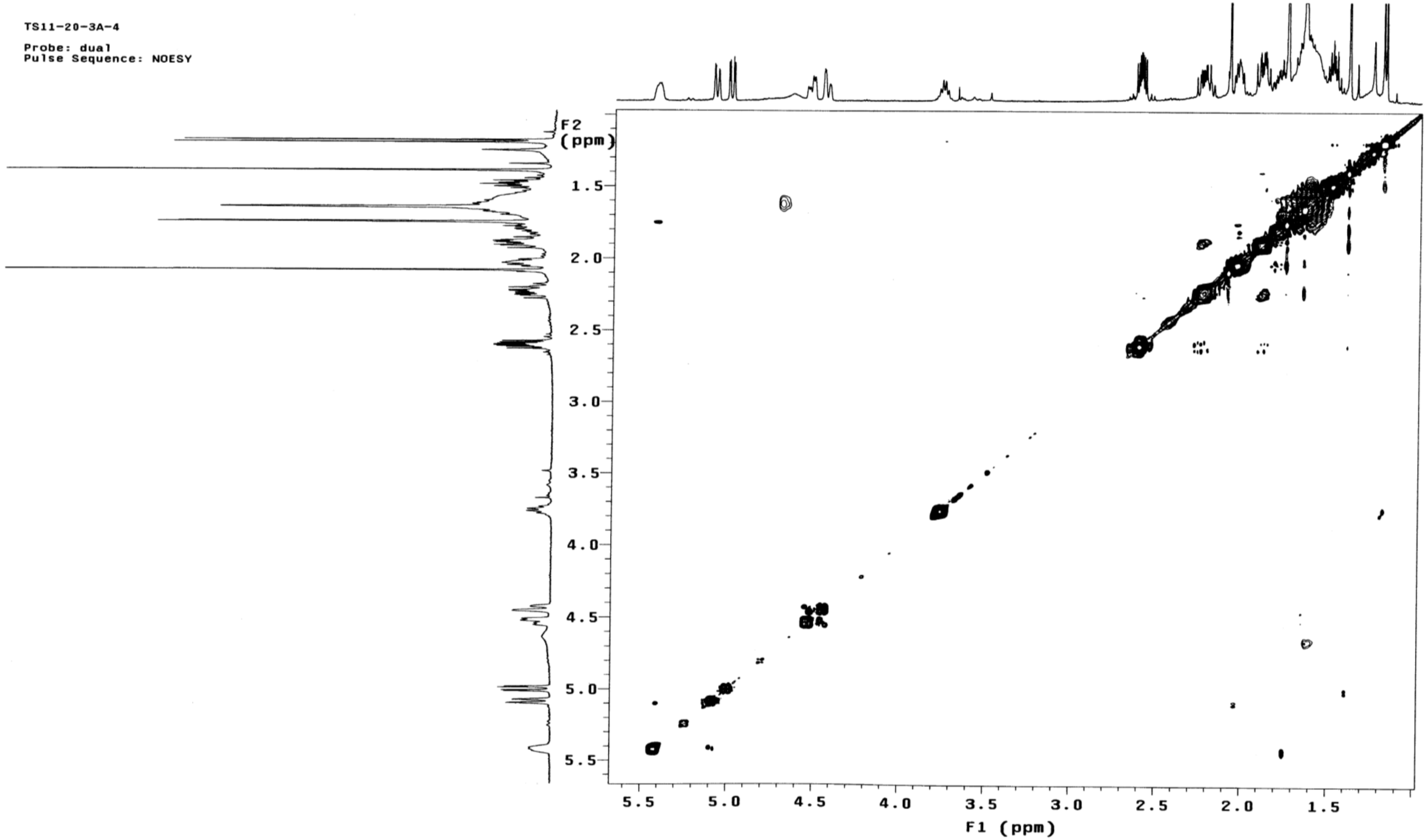

Figure S7. HRESIMS spectrum of secocrassumol.

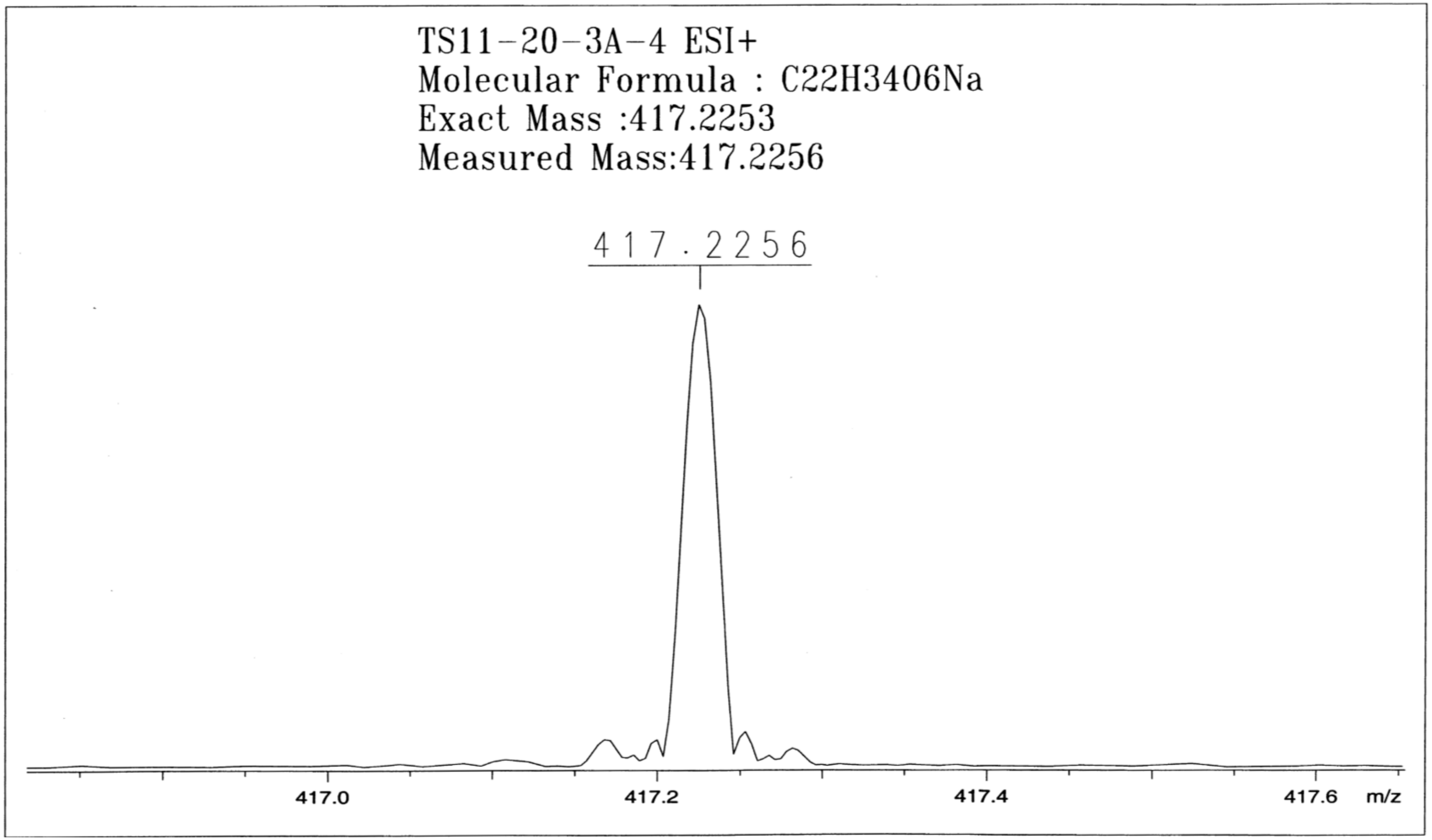

© 2014 by the authors; licensee MDPI, Basel, Switzerland. This article is an open access article distributed under the terms and conditions of the Creative Commons Attribution license (http://creativecommons.org/licenses/by/4.0/).
